# Motivational Design for Web-Based Instruction in Health Professions Education: Protocol for a Systematic Review and Directed Content Analysis

**DOI:** 10.2196/42681

**Published:** 2022-11-09

**Authors:** Adam Gavarkovs, Rashmi A Kusurkar, Kulamakan Kulasegaram, Jeff Crukley, Erin Miller, Melanie Anderson, Ryan Brydges

**Affiliations:** 1 Institute of Health Policy, Management and Evaluation University of Toronto Toronto, ON Canada; 2 The Wilson Centre University of Toronto/University Health Network Toronto, ON Canada; 3 Research in Education Amsterdam UMC Vrije Universiteit Amsterdam Amsterdam Netherlands; 4 LEARN! Research Institute for Learning and Education Faculty of Psychology and Education VU University Amsterdam Amsterdam Netherlands; 5 Department of Family and Community Medicine Temerty Faculty of Medicine University of Toronto Toronto, ON Canada; 6 Department of Speech-Language Pathology Temerty Faculty of Medicine University of Toronto Toronto, ON Canada; 7 Data Science and Statistics Toronto, ON Canada; 8 School of Physical Therapy Faculty of Health Sciences Western University London, ON Canada; 9 University Health Network Toronto, ON Canada; 10 Department of Medicine Temerty Faculty of Medicine University of Toronto Toronto, ON Canada

**Keywords:** medical education, nursing education, e-learning, serious games, instructional design, motivation, health care, health professional, professional education, digital learning, web-based learning

## Abstract

**Background:**

Web-based instruction plays an essential role in health professions education (HPE) by facilitating learners’ interactions with educational content, teachers, peers, and patients when they would not be feasible in person. Within the unsupervised settings where web-based instruction is often delivered, learners must effectively self-regulate their learning to be successful. Effective self-regulation places heavy demands on learners’ motivation, so effective web-based instruction must be designed to instigate and maintain learners’ motivation to learn. Models of motivational design integrate theories of motivation with design strategies intended to create the conditions for motivated engagement. Teachers can use such models to develop their procedural and conceptual knowledge in ways that help them design motivating instruction in messy real-world contexts. Studies such as randomized controlled trials (RCTs) and other quasi-experimental designs that compare different motivational design strategies play a critical role in advancing models of motivational design. Synthesizing the evidence from those studies can identify effective strategies and help teachers and researchers understand the mechanisms governing why strategies work, for whom, and under what circumstances.

**Objective:**

The planned review aims to analyze how studies comparing motivational design strategies for web-based instruction in HPE support and advance models of motivational design by (1) controlling for established risks to internal validity, (2) leveraging authentic educational contexts to afford ecological validity, (3) drawing on established theories of motivation, (4) investigating a wide breadth of motivational constructs, and (5) analyzing mediators and moderators of strategy effects.

**Methods:**

The planned review will use database searching, registry searching, and hand searching to identify studies comparing motivational design strategies for web-based instruction, delivered to learners in HPE. Studies will be considered from 1990 onward. Two team members will independently screen studies and extract data from the included studies. During extraction, we will record information on the design characteristics of the studies, the theories of motivation they are informed by, the motivational constructs they target, and the mediators and moderators they consider.

**Results:**

We have executed our database and registry searches and have begun screening titles and abstracts.

**Conclusions:**

By appraising the characteristics of studies that have focused on the motivational design of web-based instruction in HPE, the planned review will produce recommendations that will ensure impactful programs of future research in this crucial educational space.

**Trial Registration:**

PROSPERO CRD42022359521; https://tinyurl.com/57chuzf6

**International Registered Report Identifier (IRRID):**

DERR1-10.2196/42681

## Introduction

Learning remotely is here to stay. Web-based instruction, which encompasses remote lectures, asynchronous interactive modules, virtual patient simulations, and serious games, plays an essential role in health professions education (HPE): it digitally mediates learners’ interactions with educational content, teachers, peers, and patients, when it would otherwise be too costly, infeasible, or impossible for in-person interactions to occur [1,2].

Learners typically access web-based instruction from remote, unsupervised settings, such as home, coffee shop, or library. Accordingly, learners often have a great deal of control in terms of *how* to engage with instruction. They can choose which learning strategies to use (eg, by taking notes in a notebook), when to revisit content (eg, returning to a previous slide), whether to access help from a peer or teacher (eg, by asking questions or leaving a comment), and how long to spend on learning. Under these conditions, learners must self-regulate their learning effectively [3]. Theoretical models of self-regulated learning (SRL) construe learning as a process whereby learners set goals for their learning and then strategically monitor and control aspects of their cognition, motivation, behavior, and environment toward attaining their goals [4]. A growing body of literature in HPE has demonstrated positive relationships between facets of SRL and academic achievement in unsupervised settings [5-7].

Effective SRL requires significant effort. Learners engaged in SRL do not learn “on autopilot” by following the directions of others or by defaulting to their usual approach to learning. Rather, they actively monitor and adapt their approach to learning as necessary [8]. Consequently, SRL relies heavily on a learner’s motivation to learn [9]. Motivation refers to the energetic force that instigates and sustains goal-directed action [10]. Several studies in HPE provide evidence for links between motivational constructs, facets of SRL, and academic achievement [6,11-16].

A learner’s motivation to learn will ebb and flow depending on situational factors such as what they are learning, with whom, where, and the challenges they face along the way [17,18]. Consequently, learners may sit down at their computer to complete web-based instruction only to find themselves less than optimally motivated. In such situations, they cannot rely on a teacher to recognize they are facing a motivational deficit, nor to help them address it. Instead, motivational support can, and should, be built into instruction itself [19].

Motivational design, a subprocess of instructional design, is a systematic, goal-directed, problem-solving process that involves (1) specifying the conditions under which learners will become and remain motivated to engage with instruction and (2) designing instruction to facilitate these conditions [20]. Models of motivational design integrate (1) an underlying theoretical account of how motivated engagement in learning unfolds with (2) a set of evidence-based strategies that teachers can use to facilitate the conditions for motivated engagement [21]. While theories of motivation describe *how* learners instigate and sustain goal-directed action, models of motivational design prescribe strategies for *how to help* instigate and sustain learners’ goal-directed action toward desirable learning outcomes [21]. For example, Keller’s attention, relevance, confidence, and satisfaction (ARCS) model of motivational design, commonly used across many educational contexts, including HPE [22,23], integrates a theory of motivation (Keller’s macro model of motivation) with an organized set of strategies targeting four key motivational conditions derived from the ARCS theory [20].

Owing to their theoretical grounding, models of motivational design can help teachers build both *procedural knowledge* regarding design strategies that can be applied when designing instruction, and *conceptual knowledge* regarding why design strategies ought to be effective, based on an underlying theoretical account of how motivated engagement in learning unfolds. We argue that with an integrated body of procedural and conceptual knowledge, teachers can more flexibly apply and adapt previously learned design strategies and invent new ones in the messy, real-world contexts of HPE [24]. Therefore, we propose that a key objective of HPE research should be to advance models of motivational design.

Many kinds of studies can advance models of motivational design [25]. “Basic science” studies conducted in highly controlled lab environments can advance our understanding of the motivational processes underpinning learning [26,27]. Single-group quantitative, qualitative, and mixed-methods studies can investigate learners’ perceptions of, and reactions to, instructional designs, to support our theoretical understanding of how certain designs operate to support motivation [28]. We propose that studies that aim to compare different motivational design strategies, including randomized controlled trials (RCTs) and other quasi-experimental designs, play an essential role in advancing models of motivational design. They uniquely afford the potential for identifying the effects of different design strategies, which can then be integrated into models of motivational design. Research comparing motivational design strategies can also investigate mediating processes and moderating factors to determine why a strategy works, for whom, and under what conditions, thus helping to test and refine the theory that underlies a model of motivational design [29]. Accordingly, motivation researchers in HPE have called for greater use of RCTs to investigate strategies to enhance learners’ motivation [29,30].

In this review, we aim to appraise studies that compare motivational design strategies for web-based instruction in HPE, to enhance the quality of future research toward refined models of motivational design. Comparative studies can advance models of motivational design when they generate high-quality evidence regarding what motivational strategies work, why, for whom, and under what circumstances. Accordingly, our review will be guided by the following research questions: (1) How well do existing studies control for established risks of bias (eg, allocating participants to different instructional designs randomly)? To afford drawing *internally valid*, causal conclusions regarding the effects of a design strategy, studies must be conducted in a manner that avoids known risks of bias [31]. For instance, Lazowski and Hulleman [32] found that quasi-experimental studies of motivational interventions reported stronger, more positive effect sizes than RCTs, suggesting that quasi-experimental studies may be subject to positive bias. (2) To what extent are existing studies conducted in authentic educational contexts? For studies to draw *ecologically valid* conclusions regarding the effects of a design strategy, they are best conducted in authentic educational contexts rather than in fabricated lab environments that do not resemble the “real world” [31,32]. For instance, findings of attenuated effects may be due to lower levels of engagement with aspects of an instructional design in an authentic versus a lab context [28,33]. (3) How frequently, and to what extent, are existing studies explicitly informed by a theory of motivation or model of motivational design? Theories of motivation and models of motivational design can serve to “organize” design strategies by associating them with motivational processes sketched out in the theory or model. Doing so permits an understanding of how the effects of a strategy relate to the underlying motivational processes sketched out in the theory or model. Further, an established theory of motivation or model of motivational design can help researchers identify potential mediating processes and moderating factors that could be the subject of investigation [29]. (4) Which motivational constructs have studies targeted with their instructional designs? Theories of motivation propose many proximal determinants of motivation, such as competence beliefs and value beliefs [29]. In models of motivational design, such constructs can be considered the *conditions* under which learners will become and remain motivated to engage with instruction, and which should be facilitated by instruction. Constructs may be influential depending on the characteristics of learners, the task, and the context in which learning takes place; therefore, it is important that teachers are able to draw on design strategies targeting a wide breadth of constructs. (5) Which hypothesized mediators or moderators of motivational design have studies operationalized or analyzed? Studies outside of HPE have demonstrated that motivational interventions can have differential effects on engagement and learning, depending on learner characteristics such as perceived competence for learning [34,35]. We will catalogue the data researchers collect on potential mediating variables (eg, self-regulated learning processes) and moderating factors (eg, baseline motivational characteristics).

We have chosen a systematic review as the most appropriate review methodology for answering our research questions, given our focus on RCTs and other quasi-experimental comparisons and our interest in appraising the quality of the included studies. Like other previous reviews, our analysis will profile each study’s conceptual foundations, intervention characteristics, and chosen study designs, rather than aggregate study outcomes [36-39].

To increase the feasibility of our review, we will restrict our focus to studies that compare design strategies targeting motivation for web-based instruction. This focus is warranted; researchers have argued that effective SRL is more critical in web-based learning environments than in other, in-person learning environments, due to their unsupervised nature [39]. Further, as studies coming out of the COVID-19 pandemic have shown, learners’ motivation may be particularly vulnerable in remote, web-based learning environments [40].

## Methods

### Overview

The protocol for this systematic review is reported in accordance with PRISMA-P (Preferred Reporting Items for Systematic Review and Meta-Analysis Protocols) [41]. However, we omit items 16 (meta-biases) and 17 (confidence in cumulative evidence), given we will not synthesize the outcomes of studies. The protocol for this systematic review has been registered (PROSPERO #CRD42022359521).

### Eligibility Criteria

#### Study Characteristics

We will consider primary studies published in English, from 1990 to 2022. We selected this range based on the review strategy adopted by the Digital Health Education Collaboration, which recently published several reviews on digital education in HPE [42]. They argued computers were rarely used for educational purposes prior to 1990. We will consider study designs, including individual RCTs, cluster RCTs, cross-over trials, and other quasi-experimental designs. Notably, protocols for *ongoing* studies are also eligible for inclusion.

#### Participants

Studies will be eligible for inclusion if their sample was limited to learners in the health professions or was included but was not limited to learners in the health professions. Learners in the health professions may be preregistration or postregistration, following the distinction made by the Digital Health Education Collaboration [42]. *Preregistration* learners are those enrolled in an educational program (eg, university degree program and vocational training program) that, upon completion, renders them eligible for a qualification permitting them to work in a health care setting under a regulated professional designation. *Postregistration* learners are those already working in a health care setting under a regulated professional designation and whose learning focuses on maintaining, updating, or broadening their existing knowledge and skills with respect to their practice discipline. Our list of eligible health professions is based on a triangulation from two sources. First, we referenced the list of regulated health professions in Ontario, Canada under the *Regulated Health Professions Act, 1991*. Then, we cross-referenced this list with the list of regulated health professions in the United Kingdom. We included all the regulated professions on either list, which are as follows: audiology; arts therapy; chiropody or podiatry; chiropractic care; dental hygiene; dental technology; dental therapy; dentistry; denturism; dietetics; hearing aid dispensing; homeopathy; massage therapy; medical laboratory technology; medical radiation technology; medicine; midwifery; naturopathy or osteopathy; nursing; occupational therapy; operating department practitioner; opticianry; optometry; orthodontic therapy; orthoptics; paramedicine; pharmacy; pharmacy technology; physiotherapy; psychotherapy; prosthetics, pedorthists, or orthotists; respiratory therapy; speech-language pathology; and traditional Chinese medicine and acupuncture. We also consider social work (which is a regulated profession in Ontario) to be an eligible health profession. Although biomedical scientists, clinical scientists, kinesiologists, and psychologists are regulated health professions, many learners in these fields do not intend to pursue a health care professional designation. Consequently, we excluded these from our list of eligible health professions.

#### Interventions

We will adopt the levels of instructional design framework proposed by Cook [43], who argued that instructional design choices can be conceptualized at three levels: the instructional medium, configuration, and strategy. An *instructional medium* refers to a mode of delivery. Examples include face-to-face instruction, paper-based instruction, and web-based instruction. We define web-based instruction as any instruction that leverages internet-based technologies to digitally mediate learners’ interactions with educational content, teachers, patients, or peers [2]. An *instructional configuration* refers to a type of instruction within a given medium that has several distinguishing features from other configurations. Examples of different web-based instructional configurations include virtual lectures, asynchronous tutorials, and web-based discussion forums. An *instructional strategy* refers to a technique employed within a given configuration that is intended to facilitate the learning process. Examples of different strategies within a virtual patient simulation on communication skills include recording a transcript of the patient interview for later reflection or asking learners to set certain goals before interacting with the virtual patient.

Within studies using the medium of web-based instruction, our inclusion criteria will require that authors evaluate the effects of an instructional configuration or strategy that explicitly targets a specific motivational construct, or motivation more generally. That is, studies will be eligible if the intervention’s effect on learning is hypothesized to occur through effects on motivation. Strategies targeting the timing of instruction (eg, before or after an in-person simulation experience) or the delivery of instruction (eg, supplemented with email reminders) will also be eligible for inclusion.

Studies will be judged ineligible if an instructional configuration or strategy does not intend to enhance or maintain learners’ motivation *to learn*, but rather enhance or maintain their motivation toward some other aim. For example, a study that evaluates how an instructional strategy impacts learners’ self-efficacy to apply a new procedure in clinical practice (versus their self-efficacy for learning more about the procedure) will be excluded from this review. We are interested in identifying evidence-based methods for designing web-based instruction to energize the process of SRL *during* instruction, not in energizing the self-regulated application of learned knowledge and skills in practice.

We will also include studies if an instructional configuration or strategy is investigated within a computer-based environment that could be made available to learners via internet-based technologies but was not done so for the study. For example, a study that investigated the motivational effects of a strategy within an instructional environment made available to learners via a CD-ROM would be eligible for inclusion, as such an environment could be readily replicated and delivered to learners via the web. By contrast, a virtual learning environment that requires a head-mounted display connected to a powerful computer would not be considered an environment that could be made available to learners via the web, and thus would not be eligible for inclusion. Finally, the device (eg, computers or smartphones) that learners use to access web-based instruction will have no bearing on study eligibility.

#### Comparators

Studies will be eligible for inclusion if they compare (1) an instructional configuration with another configuration, (2) an instructional strategy with another strategy while holding the configuration constant, or (3) an instructional strategy with the absence of the strategy while holding the configuration constant. Cook [43] argued that comparisons between configurations (eg, a virtual lecture versus an asynchronous interactive module) are inherently confounded given the many points of differentiation, making it nearly impossible to connect configuration features to any differences across outcomes. Consequently, such comparisons are less informative than comparisons at the strategy level, which feature a single point of differentiation. However, our primary interest is in mapping the literature to date, so both sorts of comparisons will be included. Further, based on prior reviews, we expect most comparisons will occur at the configuration level [44].

#### Outcomes

Similar to the meta-analysis of motivation interventions in education by Lazowski and Hulleman [32], studies will be eligible for inclusion if they assess the effect of an instructional configuration or strategy on a learner outcome, including motivation, SRL, and achievement outcomes. Motivational outcomes include self-reports regarding specific motivational constructs or of motivation more generally. SRL outcomes are highly varied; based on established models of SRL [45] and prior reviews [46,47], SRL outcomes may relate to goals (including goal level and goal content), metacognitive processes (including goal setting, planning, self-monitoring, self-control, self-judgements, and self-reactions, which may relate to aspects of cognition, motivation, emotion, behavior, or the environment), cognitive strategy use (including rehearsal, organization, and elaboration strategies, or any other procedures a learner uses to control how they process task-relevant information), and resource management (including effort regulation, persistence, time management, environmental structuring, help seeking, peer collaboration, or any other procedures one uses to control their external environment or their internal environment, including their motivation and emotion). In HPE, the related construct of *engagement* has been conceptualized and operationalized to encompass a broad range of SRL processes. For example, engagement has been framed as having an experiential dimension (ie, reflecting a learner’s subjective experience while playing a game) and a behavioral dimension (ie, reflecting a learner’s time on task) [44]. From an SRL perspective [48], experiential engagement could map onto several motivational constructs, whereas behavioral engagement maps to persistence. Finally, studies are eligible if they collect any available achievement measure (eg, retention or transfer and course grades), assessed at any time (ie, immediately after instruction or delayed). Studies that only include non–learner outcomes (eg, instructor satisfaction and cost) will not be eligible for inclusion, as we do not consider these studies to be investigations of designs targeting *learners’* motivation to learn.

### Information Sources

#### Database Searching

Relevant studies will be identified by searching the following databases: MEDLINE, Embase, Emcare, PsycINFO, ERIC, and Web of Science. Articles addressing the education or training of each health profession appear in the journals of these respective fields. For this reason, databases with significant coverage of the medical, nursing, allied health, as well as education literature were selected, with the addition of the multidisciplinary database platform Web of Science. These databases are also broadly consistent with those selected for similar reviews [32,44,49]. Our search strategy was developed by a health sciences librarian in collaboration with subject specialists and informed by prior reviews [32], using MEDLINE initially to assess the quality and quantity of our search returns. The search strategy was then adapted and applied to the other databases. Categories of terms included those related to learners in eligible health professions, web-based instruction, and motivation. Unlike the review by Lazowski and Hulleman [32], we did not include theories of motivation in our search terms, as theory use was not a criterion for inclusion. Further, we did not include specific motivational constructs (eg, value, relevance, confidence, and interest) in our search terms, as we expected this would greatly increase the number of nonrelevant studies required to screen, as many motivational constructs are common words used in nonmotivational contexts. Rather, we assume that any study targeting motivation and referencing a specific motivational construct will also mention motivation, and thus will be covered in our search. Our search strategy for MEDLINE can be found in Multimedia Appendix 1.

#### Registry Searching

Relevant studies will be identified by searching Open Science Framework Registries.

#### Hand Searching

Studies will also be identified by hand searching the reference lists of previous systematic reviews related to web-based instruction in HPE [38,44,49-58].

#### Reference Searching

The reference lists of the included studies will also be screened for additional studies.

### Study Records

#### Data Management

All records identified through database and hand searching will be managed and screened using Covidence web-based software. After title and abstract screening, the full texts of the included studies will be uploaded for screening and, if necessary, data extraction.

#### Selection

The titles and abstracts of all records identified through database and hand searching will be independently screened by 2 team members, who will be blinded to each other’s decisions. Team members will periodically meet to review conflicts, identify any systematic reasons for conflicts, and come to decisions regarding how to handle these issues. With these decisions in mind, conflicts will then be resolved by one team member not involved in the initial decision. The same process will occur for full-text screening. Reason for exclusion at the full-text screening stage will be documented and reported in a PRISMA (Preferred Reporting Items for Systematic Review and Meta-Analysis) flow diagram. Percent agreement at the abstract and full-text screening stages will be calculated.

#### Risk of Bias Assessment

The risk of bias for the included studies will be independently rated by 2 team members using the Cochrane Effective Practice and Organization of Care standard risk of bias criteria [59]. The 9 criteria involved in a risk of bias assessment include the following: random sequence generation, allocation concealment, similar baseline outcome measures, similar baseline characteristics, incomplete outcome data, blind assessment of outcomes, protection against contamination, selective outcome reporting, and other risks of bias. Each criterion will be given a rating of “low risk,” “high risk,” or “unclear risk” at the study level. Team members will be blinded to each other’s rating. Team members will periodically meet to review conflicts, identify any systematic reasons for conflicts, come to decisions regarding how to handle these issues, and resolve conflicts in ratings. Percent agreement for risk of bias ratings will be calculated.

#### Data Extraction and Synthesis

Data will be extracted and synthesized using a directed content analysis with each individual study as the unit of analysis [60]. We will use content analysis to systematically code the content of each article into categories for the purpose of identifying patterns in the data [60,61]. We will code deductively, meaning we will use existing theory or prior research as a foundation for developing our initial coding categories [60]. Our coding scheme will likely not remain static, given we will iteratively adapt it when relevant data are not congruent with existing categories [61]. Study data will be independently extracted by 2 team members using a comprehensive extraction and coding tool, developed through a consultation of the theoretical and empirical literature. The extraction and coding tool will be piloted and, as necessary, updated by having 2 team members independently extract data from a few studies and comparing their results. Team members will be blinded during the extraction process. Team members will periodically meet to review variability in the extracted data, identify any systematic reasons for this variability, and decide how to handle these issues. Primary study authors will be contacted in the case of unclear or missing data. Percept agreement for extracted items will be calculated.

Our extraction and coding tool will facilitate the collection of the following data items: study title, first author, publication year, geographic location in which the study was completed, study design, health profession of participants, training status of participants, sample size, topics of instruction, length of instruction, setting in which instruction was delivered to participants, device on which instruction was accessed, technology used to deliver instruction (eg, internet or CD-ROM), instructional configuration, instructional strategy (if relevant), theory of motivation used to inform the configuration or strategy, motivational constructs targeted by a configuration or strategy, definition of the constructs (if applicable), other learning processes targeted by a configuration or strategy (eg, cognitive processes), comparison, moderators, outcomes (including hypothesized mediating variables), and moderator or outcome measures.

Two items relevant to our research questions are the theory of motivation used and the motivational constructs targeted by a configuration or strategy. We have developed a list of 7 of the most established theories of motivation in education, to be used as our initial codes, as follows: (1) expectancy-value theory [62]; (2) achievement goal theory [63]; (3) self-determination theory [64,65]; (4) social cognitive theory [66,67]; (5) attribution theory [68]; (6) control-value theory [69,70]; and (7) the Keller macro model of motivation and performance (underpinning the ARCS model of motivational design) [20,71]

Based on this list of theories, we also developed an initial list of motivational constructs, which comprises the following: (1) achievement goal orientations; (2) competence beliefs (including confidence, self-efficacy, action-control expectancies, outcome expectancies, action-outcome expectancies, control of learning beliefs, and expectancies for success); (3) value beliefs (including relevance, perceived instrumentality, task value, extrinsic value, utility value, attainment value, and cost); (4) interest (also curiosity and attention); (5) outcome attributions; (6) the self-determination theory taxonomy of motivation (intrinsic motivation, extrinsic motivation, external regulation, introjected regulation, identified regulation, integrated regulation, autonomous motivation, and controlled motivation); and (7) basic psychological needs satisfaction or frustration (including feelings of autonomy, competence, and relatedness).

These codes are by no means restrictive; we anticipate that they will inductively grow and change through the data extraction process. The results of our deductive content analysis will be presented in tabular and graphical form, representing the frequency of different study characteristics, the frequency with which different motivational constructs have been targeted in the literature, including as mediators and moderators, and the theories of motivation that have informed these studies. We will also present stratified results by type of health professional, participants’ training status, and study characteristics. These tabular and graphical presentations will be accompanied by narratively presented exemplars of strategies targeting different constructs.

## Results

As of September 2022, we have completed our database searches (executed on August 2, 2022) and registry searches (executed on September 15, 2022) and have begun hand searching. Our initial search yielded 10,590 studies. We selected a purposive sample of 30 studies for team members to practice screening. Following practice, we began screening titles and abstracts. We aim to complete screening by the end of 2022.

## Discussion

### Overview

Through conducting this review, we expect to produce a list of understudied or poorly studied conceptual foci to support the growth of a robust evidence base in motivational design, and to provide guidance regarding methodological advancements in future studies of motivational design (eg, greater use of moderation analyses). By establishing a foundation to guide future theory-based research in this area, our review will provide more fertile grounds for future knowledge syntheses that include other sources of evidence (eg, qualitative studies) and that focus on understanding mechanisms of motivational design (eg, realist reviews).

Although previous reviews have focused on motivational design features of web-based instruction in HPE [38,44,49-58], none have sought to (1) achieve the specific goal of using existing evidence to refine models of motivational design, (2) propose which types of evidence will be required to meet this goal, (3) identify the study designs that can generate such evidence (eg, studies comparing motivational design strategies), and (4) appraise the degree to which studies have generated such evidence. Thus, the value of our review lies in its ability to appraise where we have been and to inform where we ought to go. We anticipate our findings will inform a program of research that includes future experimental studies, qualitative studies, and additional knowledge syntheses.

### Conclusion

In his book on motivational design, Keller [20] posed the question “Is motivation like a boulder – stable and unwavering, or a pile of dry leaves – unstable and in flux?” The answer appears to be *both*, depending on the level of generality at which motivation is assessed [18]. Most learners in the health professions report being highly motivated to improve their knowledge and skills [72], a consistent finding across disciplines that has likely perpetuated a belief that learners are *always* motivated to learn. However, from situation to situation, learners in HPE likely experience fluctuations in their motivation, depending on what they are learning, the context in which learning takes place, and the challenges they face along the way [18]. Viewing motivation at the situational level demands that we understand ways of designing web-based instruction to enhance and maintain learners’ motivation. Through this systematic review, we aim to support future research regarding the motivational design of web-based instruction in HPE by appraising the characteristics of RCTs and other quasi-experimental comparisons that have been conducted to date. We believe this new era of remote learning demands that we set a strong foundation for researchers to generate the highest quality evidence toward ensuring HPE learners flourish rather than languish when learning online.

## Appendix

Multimedia Appendix 1 MEDLINE search strategy.

## Abbreviations

ARCS: attention, relevance, confidence, and satisfaction
HPE: health professions education
PRISMA: Preferred Reporting Items for Systematic Review and Meta-Analysis
PRISMA-P: Preferred Reporting Items for Systematic Review and Meta-Analysis Protocols
RCT: randomized controlled trial
SRL: self-regulated learning

## References

[1] Cook DA (2014). The value of online learning and MRI: finding a niche for expensive technologies. Med Teach.

[2] Ellaway R (2018). Technology-enhanced learning. Understanding Medical Education: Evidence, Theory, and Practice. 3rd ed.

[3] Kizilcec RF, Pérez-Sanagustín M, Maldonado JJ (2017). Self-regulated learning strategies predict learner behavior and goal attainment in Massive Open Online Courses. Computers & Education.

[4] Pintrich P (2000). The role of goal orientation in self-regulated learning. Handbook of Self-Regulation.

[5] Gandomkar R, Yazdani K, Fata L, Mehrdad R, Mirzazadeh A, Jalili M, Sandars J (2020). Using multiple self-regulated learning measures to understand medical students' biomedical science learning. Med Educ.

[6] Song H, Kalet A, Plass J (2015). Interplay of prior knowledge, self-regulation and motivation in complex multimedia learning environments. Journal of Computer Assisted Learning.

[7] Brydges R, Manzone J, Shanks D, Hatala R, Hamstra SJ, Zendejas B, Cook DA (2015). Self-regulated learning in simulation-based training: a systematic review and meta-analysis. Med Educ.

[8] Butler DL, Winne PH (2016). Feedback and Self-Regulated Learning: A Theoretical Synthesis. Review of Educational Research.

[9] Pintrich PR (1999). The role of motivation in promoting and sustaining self-regulated learning. International Journal of Educational Research.

[10] Schunk D, Meece J, Pintrich P (2015). Motivation in Education: Theory, Research, and Applications. 4th ed.

[11] Cook DA, Beckman TJ, Thomas KG, Thompson WG (2009). Measuring motivational characteristics of courses: Applying Kellerʼs instructional materials motivation survey to a web-based course. Academic Medicine.

[12] Kusurkar RA, Croiset G, Galindo-Garré F, Ten Cate O (2013). Motivational profiles of medical students: association with study effort, academic performance and exhaustion. BMC Med Educ.

[13] Kusurkar RA, Ten Cate TJ, Vos CMP, Westers P, Croiset G (2013). How motivation affects academic performance: a structural equation modelling analysis. Adv Health Sci Educ Theory Pract.

[14] Isik U, Wilschut J, Croiset G, Kusurkar RA (2018). The role of study strategy in motivation and academic performance of ethnic minority and majority students: a structural equation model. Adv Health Sci Educ Theory Pract.

[15] Orsini CA, Binnie VI, Jerez OM (2019). Motivation as a Predictor of Dental Students' Affective and Behavioral Outcomes: Does the Quality of Motivation Matter?. Journal of Dental Education.

[16] Orsini CA, Binnie VI, Tricio JA (2018). Motivational profiles and their relationships with basic psychological needs, academic performance, study strategies, self-esteem, and vitality in dental students in Chile. J Educ Eval Health Prof.

[17] Cleary TJ, Dong T, Artino AR (2015). Examining shifts in medical students' microanalytic motivation beliefs and regulatory processes during a diagnostic reasoning task. Adv Health Sci Educ Theory Pract.

[18] Vallerand R (2000). Deci and Ryan's self-determination theory: a view from the hierarchical model of intrisnic and extrinsic motivation. Psychol Inq.

[19] Cook DA, Gas BL, Farley DR, Lineberry M, Naik ND, Cardenas Lara FJ, Artino AR (2019). Influencing mindsets and motivation in procedural skills learning: Two randomized studies. J Surg Educ.

[20] Keller J (2010). Motivational Design for Learning and Performance: The ARCS Model Approach.

[21] Reigeluth C (1999). What is instructional-design theory and how is it changing?. Instructional-Design Theories and Models: A New Paradigm of Instructional Theory.

[22] Li K, Keller JM (2018). Use of the ARCS model in education: A literature review. Computers & Education.

[23] Lounsbery JL, Reidt SL, Pittenger AL (2020). Motivational design to grab attention, establish relevance, build confidence, and achieve satisfaction about evidence‐based medicine for pharmacists. J Am Coll Clin Pharm.

[24] Hatano G, Inagaki K (1986). Two courses of expertise. Child Development and Education in Japan.

[25] Cook DA (2009). The failure of e-learning research to inform educational practice, and what we can do about it. Med Teach.

[26] Deci EL, Eghrari H, Patrick BC, Leone DR (1994). Facilitating internalization: the self-determination theory perspective. J Pers.

[27] Sansone C, Weir C, Harpster L, Morgan C (1992). Once a boring task always a boring task? Interest as a self-regulatory mechanism. Journal of Personality and Social Psychology.

[28] Brisson BM, Hulleman CS, Häfner I, Gaspard H, Flunger B, Dicke A, Trautwein U, Nagengast B (2020). Who sticks to the instructions—and does it matter? Antecedents and effects of students’ responsiveness to a classroom-based motivation intervention. Z Erziehungswiss.

[29] Cook DA, Artino AR (2016). Motivation to learn: an overview of contemporary theories. Med Educ.

[30] Kusurkar R (2012). Self-determination theory in health professions education. The Handbook of Self-Determination Theory.

[31] Nezu A, Nezu C (2008). The "devil is in the details": recognizing and dealing with threats to validity in randomized controlled trials. Evidence-Based Outcome Research: A Practical Guide to Conducting Randomized Controlled Trials for Psychosocial Interventions.

[32] Lazowski RA, Hulleman CS (2016). Motivation interventions in education. Review of Educational Research.

[33] Hulleman CS, Cordray DS (2009). Moving From the Lab to the Field: The Role of Fidelity and Achieved Relative Intervention Strength. Journal of Research on Educational Effectiveness.

[34] Durik AM, Shechter OG, Noh M, Rozek CS, Harackiewicz JM (2014). What if I can’t? Success expectancies moderate the effects of utility value information on situational interest and performance. Motiv Emot.

[35] Shechter OG, Durik AM, Miyamoto Y, Harackiewicz JM (2011). The role of utility value in achievement behavior: the importance of culture. Pers Soc Psychol Bull.

[36] Ash T, Agaronov A, Young T, Aftosmes-Tobio A, Davison KK (2017). Family-based childhood obesity prevention interventions: a systematic review and quantitative content analysis. Int J Behav Nutr Phys Act.

[37] Cook DA, Brydges R, Zendejas B, Hamstra SJ, Hatala R (2013). Technology-Enhanced Simulation to Assess Health Professionals. Academic Medicine.

[38] Tudor Car L, Kyaw BM, Teo A, Fox TE, Vimalesvaran S, Apfelbacher C, Kemp S, Chavannes N (2022). Outcomes, measurement instruments, and their validity evidence in randomized controlled trials on virtual, augmented, and mixed reality in undergraduate medical education: Systematic mapping review. JMIR Serious Games.

[39] Alvarez RP, Jivet I, Perez-Sanagustin M, Scheffel M, Verbert K (2022). Tools designed to support self-regulated learning in online learning environments: A systematic review. IEEE Trans. Learning Technol.

[40] McFadden S, Guille S, Daly-Lynn J, O'Neill B, Marley J, Hanratty C, Shepherd P, Ramsey L, Breen C, Duffy O, Jones A, Kerr D, Hughes C (2022). Academic, clinical and personal experiences of undergraduate healthcare students during the COVID-19 pandemic: A prospective cohort study. PLoS One.

[41] Moher D, Shamseer L, Clarke M, Ghersi D, Liberati A, Petticrew M, Shekelle P, Stewart LA, PRISMA-P Group (2015). Preferred reporting items for systematic review and meta-analysis protocols (PRISMA-P) 2015 statement. Syst Rev.

[42] Car J, Carlstedt-Duke J, Tudor Car L, Posadzki P, Whiting P, Zary N, Atun R, Majeed A, Campbell J, Digital Health Education Collaboration (2019). Digital Education in Health Professions: The Need for Overarching Evidence Synthesis. J Med Internet Res.

[43] Cook DA (2005). The research we still are not doing: An agenda for the study of computer-based learning. Acad Med.

[44] Maheu-Cadotte M, Cossette S, Dubé V, Fontaine G, Lavallée A, Lavoie P, Mailhot T, Deschênes M-F (2021). Efficacy of serious games in healthcare professions education: A systematic review and meta-analysis. Simul Healthc.

[45] Panadero E (2017). A review of self-regulated learning: Six models and four directions for research. Front Psychol.

[46] Dent AL, Koenka AC (2015). The relation between self-regulated learning and academic achievement across childhood and adolescence: A meta-analysis. Educ Psychol Rev.

[47] Sitzmann T, Ely K (2011). A meta-analysis of self-regulated learning in work-related training and educational attainment: what we know and where we need to go. Psychol Bull.

[48] Butler DL, Cartier SC (2022). Promoting effective task interpretation as an important work habit: A key to successful teaching and learning. Teachers College Record.

[49] Cook DA, Levinson AJ, Garside S, Dupras DM, Erwin PJ, Montori VM (2010). Instructional design variations in internet-based learning for health professions education: a systematic review and meta-analysis. Acad Med.

[50] Wang R, DeMaria S, Goldberg A, Katz D (2016). A systematic review of serious games in training health care professionals. Simul Healthc.

[51] Gentry SV, Gauthier A, L'Estrade Ehrstrom B, Wortley D, Lilienthal A, Tudor Car L, Dauwels-Okutsu S, Nikolaou CK, Zary N, Campbell J, Car J (2019). Serious gaming and gamification education in health professions: Systematic review. J Med Internet Res.

[52] Fontaine G, Cossette S, Maheu-Cadotte M, Mailhot T, Deschênes M-F, Mathieu-Dupuis G, Côté J, Gagnon M, Dubé V (2019). Efficacy of adaptive e-learning for health professionals and students: a systematic review and meta-analysis. BMJ Open.

[53] Silva RDOS, Pereira AM, Araújo DCSAD, Rocha KSS, Serafini MR, de Lyra Jr DP (2021). Effect of digital serious games related to patient care in pharmacy education: A systematic review. Simulation & Gaming.

[54] Min A, Min H, Kim S (2022). Effectiveness of serious games in nurse education: A systematic review. Nurse Educ Today.

[55] Szeto MD, Strock D, Anderson J, Sivesind TE, Vorwald VM, Rietcheck HR, Weintraub GS, Dellavalle RP (2021). Gamification and game-based strategies for dermatology education: Narrative review. JMIR Dermatol.

[56] Sipiyaruk K, Hatzipanagos S, Reynolds PA, Gallagher JE (2021). Serious games and the COVID-19 pandemic in dental education: An integrative review of the literature. Computers.

[57] Arruzza E, Chau M (2021). A scoping review of randomised controlled trials to assess the value of gamification in the higher education of health science students. J Med Imaging Radiat Sci.

[58] Xu Y, Lau Y, Cheng LJ, Lau ST (2021). Learning experiences of game-based educational intervention in nursing students: A systematic mixed-studies review. Nurse Educ Today.

[59] Suggested risk of bias criteria for EPOC reviews. Cochrane Effective Practice and Organisation of Care (EPOC).

[60] Hsieh H, Shannon SE (2005). Three approaches to qualitative content analysis. Qual Health Res.

[61] Finfgeld-Connett D (2013). Use of content analysis to conduct knowledge-building and theory-generating qualitative systematic reviews. Qualitative Research.

[62] Eccles JS, Wigfield A (2020). From expectancy-value theory to situated expectancy-value theory: A developmental, social cognitive, and sociocultural perspective on motivation. Contemporary Educational Psychology.

[63] Urdan T, Kaplan A (2020). The origins, evolution, and future directions of achievement goal theory. Contemporary Educational Psychology.

[64] Ryan RM, Deci EL (2020). Intrinsic and extrinsic motivation from a self-determination theory perspective: Definitions, theory, practices, and future directions. Contemporary Educational Psychology.

[65] Ten Cate ThJ, Kusurkar RA, Williams GC (2011). How self-determination theory can assist our understanding of the teaching and learning processes in medical education. AMEE guide No. 59. Med Teach.

[66] Schunk DH, DiBenedetto MK (2020). Motivation and social cognitive theory. Contemporary Educational Psychology.

[67] Artino AR (2012). Academic self-efficacy: from educational theory to instructional practice. Perspect Med Educ.

[68] Graham S (2020). An attributional theory of motivation. Contemporary Educational Psychology.

[69] Pekrun R (2006). The control-value theory of achievement emotions: Assumptions, corollaries, and implications for educational research and practice. Educ Psychol Rev.

[70] Artino AR, Holmboe ES, Durning SJ (2012). Control-value theory: using achievement emotions to improve understanding of motivation, learning, and performance in medical education: AMEE Guide No. 64. Med Teach.

[71] Keller JM (1987). Development and use of the ARCS model of instructional design. Journal of Instructional Development.

[72] Wouters A, Croiset G, Schripsema NR, Cohen-Schotanus J, Spaai GWG, Hulsman RL, Kusurkar RA (2017). A multi-site study on medical school selection, performance, motivation and engagement. Adv Health Sci Educ Theory Pract.

