# Supplementary material for: Motivational Design for Web-Based Instruction in Health Professions Education: Protocol for a Systematic Review and Directed Content Analysis
Source: JMIR Res Protoc. 2022 Nov 9;11(11):e42681. doi: 10.2196/42681 (PMC9685516; doi:10.2196/42681)
Supplement: Multimedia Appendix 1 [file resprot_v11i11e42681_app1.docx]

**Supplementary File 1: MEDLINE Search Strategy**

Database(s): Ovid MEDLINE(R) ALL 1946 to July 28, 2022
Search Strategy:

| **#** | **Searches** | **Results** |
| --- | --- | --- |
| 1 | Computer-Assisted Instruction/ | 12433 |
| 2 | exp video-audio media/ | 37893 |
| 3 | (elearn* or e-learn*).mp. | 4351 |
| 4 | internet-based.mp. | 10999 |
| 5 | web-based.mp. | 39260 |
| 6 | virtual.mp. | 83913 |
| 7 | online.mp. | 186637 |
| 8 | computer-based.mp. | 15076 |
| 9 | net-based.mp. | 516 |
| 10 | webinar*.mp. | 1361 |
| 11 | video?.mp. | 176937 |
| 12 | (recorded or recording?).mp. | 814627 |
| 13 | learning management system?.mp. | 440 |
| 14 | digital??.mp. | 191117 |
| 15 | (Blackboard Learn or CERTPOINT or D2L or Desire2Learn or Brightspace or eCollege or Edmodo or EduNxt or Engrade or GlobalScholar or (Glow adj20 Scott*) or HotChalk or Kahoot or Kannu or SAP or Skillsoft or Spongelab or SuccessFactors or "SumTotal Systems" or Taleo or (Teams adj20 microsoft) or Uzity or aTutor or Chamilo or Claroline or Canvas or eFront or ILIAS or LAMS or "LON CAPA" or Moodle or "Open edX" or OLAT or OpenOLAT or Sakai or SWAD or WeBWorK or "e khool" or CallidusCloud or "Cornerstone OnDemand" or DoceboLMS or eFront or EthosCE or "Google Classroom?" or Grovo or "Growth Engineering" or "Halogen Software" or "Inquisiq R3" or "Learning Suite" or itslearning or Kannu or OpenLearning or Udutu or "ANGEL Learning" or Click2Learn or CourseInfo or "Learn com" or Elluminate or PeopleSoft or "Plateau Systems" or Softscape or SuccessFactors or WebCT or Instructure or Oracle or Docent or Zoom or coursera or futurelearn or iversity or "khan academy" or udemy or swayam or "mit ocw" or openclassroom?).mp. [list of LMS and online learning platforms] | 22141 |
| 16 | edutech.mp. | 1 |
| 17 | (synchronous or asynchronous).mp. | 50043 |
| 18 | webcast*.mp. | 1782 |
| 19 | screencast*.mp. | 61 |
| 20 | (screenshar* or screen-shar*).mp. | 62 |
| 21 | (mlearn* or m-learn*).mp. | 112 |
| 22 | web-conferenc*.mp. | 160 |
| 23 | computer simulation*.mp. | 220954 |
| 24 | cd-rom.mp. | 1757 |
| 25 | (vhs? or dvd?).mp. | 3761 |
| 26 | or/1-25 | 1664884 |
| 27 | Motivation/ | 76677 |
| 28 | motivat*.ti,kf. | 29433 |
| 29 | (motivat* adj4 (student* or learner* or staff* or educat* or trainee* or instructee* or user? or pupil* or mentee* or tutee* or antendee* or participant*)).ab. | 9559 |
| 30 | or/27-29 | 93359 |
| 31 | 26 and 30 | 9281 |
| 32 | (teach* or educat* or academic).jw. | 177551 |
| 33 | education, predental/ or education, premedical/ or exp education, professional/ or exp inservice training/ or exp schools, health occupations/ or professional development/ | 368507 |
| 34 | exp Students, Health Occupations/ | 82728 |
| 35 | patient simulation/ | 5437 |
| 36 | or/32-35 | 495448 |
| 37 | exp Teaching/ | 91764 |
| 38 | exp curriculum/ | 95052 |
| 39 | clinical competence/ | 102807 |
| 40 | professional competence/ | 25023 |
| 41 | pedagog*.mp. | 11526 |
| 42 | ed.fs. | 296262 |
| 43 | (student * or learner* or learned or learns or teach* or educat* or instruct* or class or classes or workshop? or module? or train??? or curricul* or upskill* or retrain??? or webinar* or elearn* or e-learn* or in-service or inservice or professional develop* or continuing professional or CPD or CME or life-long-learn* or lifelong* learn* or certificate program? or certification?).ti,kw,hw. | 1081616 |
| 44 | educat*.mp. | 1161674 |
| 45 | competen*.ti,kf,hw. | 166344 |
| 46 | or/37-45 | 1604270 |
| 47 | exp health personnel/ | 588112 |
| 48 | exp health occupations/ | 1818267 |
| 49 | (health* adj4 (personnel or occupation* or profession*)).mp. | 395823 |
| 50 | (medic* or physician* or doctor* or p?ediatric* or allergist* or anesthesiologist* or dermatologist* or radiologist* or general practitioner* or geneticist* or neurologist* or obstetrician* or gyn?ecologist* or ophthalmologist* or pathologist* or oncologist* or urologist* or surgeon* or nurs* or therapist* or dent* or pharmac* or allied health* or physiotherapist* or psychiatrist* or psychologist* or audiolog* or chirop* or podiatr* or chiropractor* or dietition* or dietetic* or hearing aid* or homeopath* or RMT or medic* laborator* or medical radiat* or radiographer* or midwif* or midwiv* or naturopath* or osteopath* or operating department* or optician* or optometr* or orthodont* or orthopt* or paramed* or prosthetic* or pedorthist* or orthotist* or respirator* or speech language or social work* or traditional chinese medic* or acupunctur*).mp.  [All the regulated professions in Ontario, Canada under the Regulated Health Professions Act, 1991 or in the United Kingdom – inclusive] | 9820935 |
| 51 | (hospital* adj4 (personnel or staff)).mp. | 99673 |
| 52 | (clinic* adj4 (personnel or staff)).mp. | 12973 |
| 53 | or/47-52 | 10540900 |
| 54 | 46 and 53 | 971777 |
| 55 | 36 or 54 | 1094695 |
| 56 | 31 and 55 | 2238 |
| 57 | limit 56 to yr="1990 -Current" | 2201 |
